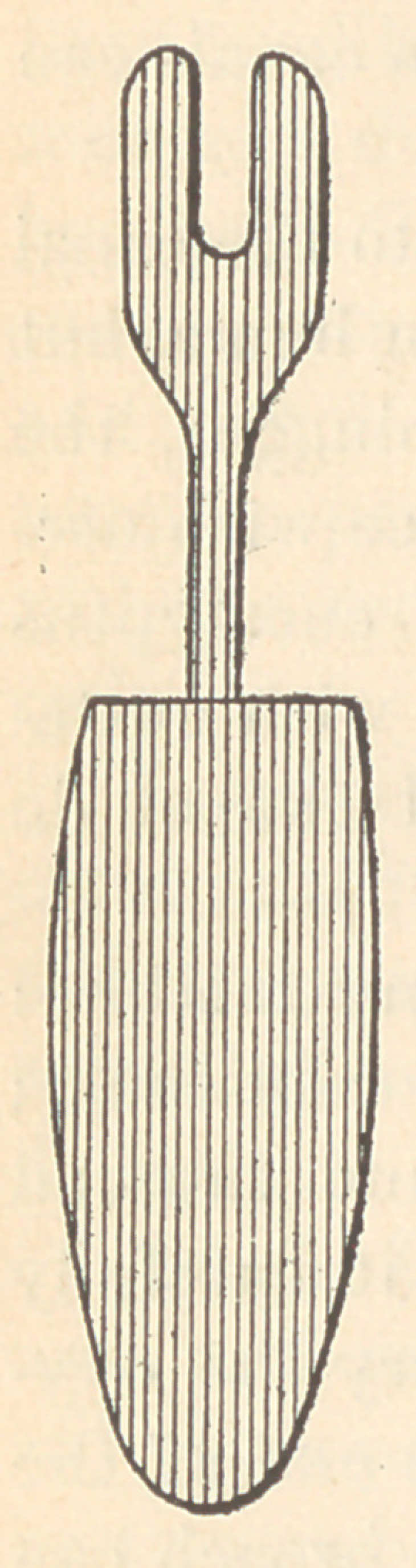# New York Odontological Society

**Published:** 1895-09

**Authors:** 

**Affiliations:** New York Odontological Society


### Reports of Society Meetings.

### NEW YORK ODONTOLOGICAL SOCIETY.

A REGULAR meeting of the New York Odontological Society was held on Tuesday evening, April 16, 1895, at the New York Academy of Medicine, No. 17 West Forty-third Street, New York City, with the President, Dr. Northrop, in the chair.

The questions for the evening's consideration were,—

1. "Which method of root-canal filling furnishes the most complete obliteration of this space?"

2. "What is the surest treatment to secure root-canal sterilization?"

### DISCUSSION.

Question 1 was first considered by a paper of Dr. S. G. Perry. (For Dr. Perry's paper, see page 527.)

Dr. Darby briefly replied that he considered oxychloride of zinc the best for this purpose, and had thought so for twenty years. He might be mistaken; if so, he did not know it.

*Dr. Louis Jack.*—The results of clinical observations appear to establish the conclusion that upon the complete obliteration of root-canals of devitalized teeth depends the prevention of after-disturbances of the peridental membranes at the apical region.

If open space remains, it will inevitably become at length occupied by organic matter, which enters by imbibition through the foramen, or by the channels of the canaliculi of the fang, which at length must acquire an infectious quality, and will sooner or later have the specific character which has been elucidated by Dr. Miller.

This consideration must also apply to all methods of root-filling, when attempts are made to occlude the apex with porous substances charged with antiseptics, as when the antisepsis terminates by the diffusion of the chemical the porous filling-material is little better than an open space. In my experience the most dangerous conditions have accompanied cotton filling in roots.

To the filling of canals where they are of good size and can be easily made accessible, such as the six front teeth, the palatal roots of upper molars, and distal roots of lower molars, I have found no substance so good for filling the apical portion of the canals as small cones of gold-foil malleted into place, the remainder of the root being filled with gutta-percha or oxychloride of zinc. For the small canals of bicuspids, the buccal canals of upper molars, and the mesial roots of lower molars, the oxychloride of zinc or chloro-percha pumped in as far as attainable have been my usual reliance.

*Dr. E. C. Kirk.*—Fill the desiccated canal with melted paraffine and pack in a gutta-percha cone of proper size and shape, avoiding excess of paraffine.

*Dr. A. W. Harlan.*—There are three substances which may be used to obliterate canal space,—wax, gutta-percha, and paraffine. All are unalterable in the root of a tooth, all comparatively easy to introduce. Not one of these materials will absorb moisture or deteriorate in its presence in such a place as a root. The roots must be dried and a solvent used to liquefy gutta-percha, wax, or paraffine. After a portion of the liquefied substance is introduced, a larger, more solid piece, attenuated or pointed, may be packed directly into the roots until they are completely filled. Force, combined with gentle heat, will be sufficient to pack the roots full of either material. I prefer gutta-percha. Teeth will not be stained by deterioration of the filling-material, and no permanent soreness will ensue in case a small quantity is forced through the apex. It will exfoliate or become encysted.

*Dr. C. N. Peirce.*—I have but a few moments to reply to your inquiries. Without doubt in my mind, gutta-percha, when properly applied or inserted, makes the most complete root-filling; but it is only mechanically so; as you know, it has no therapeutic influence. With zinc chloride or some other good antiseptic in advance of it, it probably cannot be excelled. Salol I have used with great satisfaction, but experience with it is of too recent a date to say what of the future.

*Dr. S. H. Guilford.*—The fact that so many substances have been used by different practitioners, with apparently equal success, makes it difficult to designate any particular one as best. Years ago gold-foil, employed by those who were really skilful in its manipulation, produced most satisfactory results. Gutta-percha and oxychloride of zinc followed, and appeared to answer their purpose perfectly, while later carefully-prepared sheep's wool furnished a record of usefulness unsurpassed by any of those that preceded it.

Large experience and length of time have, I think, about decided that for ease of introduction, perfect filling of the space, non-shrinkage, and unchangeableness, together with its aseptic qualities, oxychloride of zinc heads the list of materials suitable for root-canal filling.

*Dr. L. D. Shepard.*—I judge you want the methods which each uses, and not theorizing. For very many years I have used the red gutta-percha, which I roll into a fine and long cone. This may be two inches long, and tapering from one-sixteenth to one thirty-second of an inch at its greatest diameter to a point. Working some chloroform into the canal, I take the cone in a pair of delicate forceps, hold it a moment or so in chloroform, so that the outside is softened or partially dissolved, and then press the cone, *cold*, into the canal. This cone, while soft and sticky on the outside, is, as a whole, stiff, elastic, and yielding, and, I am quite sure, "obliterates the space."

*Dr. John S. Marshall.*—In my judgment, chloro-percha and gutta-percha points most perfectly obliterate this space.

*Dr. McQuillan.*—I consider, where care is used not to force the material through the apical foramen, that the oxychloride of zinc is the very best for filling the canal.

*Dr. J. B. Littig.*—The best method of root-canal filling is the one wherein we place in the root-canal a few fibres of cotton, twisted on a smooth broach and saturated with oxychloride of zinc.

*Dr. J. Y. Crawford.*—I believe the very best results in filling root-canals can be accomplished by thoroughly drying, and, when exudates have ceased from the apical region, trim a small, fine needle of orange or some other wood made sterile by proper treatment, and around the tiny end of which roll a small quantity of Abbey's soft gold-foil, and then top it into the apical third of the canal, after which the remaining portion of the canal can be filled with any of the reputable root-fillings desired, except tin, lead, and amalgam. The objections to the tin, lead, and amalgam are that they are factors in the discoloration of pulpless teeth when placed in the roots. I believe by this means that space in the canals can be most effectually obliterated.

*Dr. G. V. Black.*—Gutta-percha cones, not started into the canal with pliers and a hit-or-miss effect made to force them home, but stuck onto the end of a properly-shaped root-canal plugger, the size having been ascertained by trial, and sent to place with certainty. The canal is to be well moistened with oil of eucalyptus first. Canals too small for this are done the best I can with gutta-percha dissolved in chloroform, but this only when I cannot do better.

*Dr. J. N. Crouse.*—I would recommend the following method: Put some oxychloride of zinc at the entrance of the root-canal; then wrap one or two thicknesses of No. 10 gold around the end of a broach, dip it in oxychloride of zinc, and push it carefully into the end of the canal, using it as a medium to carry the oxychloride. The gold should be lapped loosely around the end of the broach, so that when it is carried into the root-canal the broach can be removed, leaving the gold and oxychloride of zinc at the end of the canal. Gold can be carried in this way to the end of the smallest canals; and by taking a watch-maker's pivot broach and drawing the temper just enough to get the blue color, you have a spring temper which I think makes the best instrument for filling in this manner. If the gold is carefully wrapped around the broach, and the oxychloride is carried ahead of it, the canal is thoroughly filled and all the air is excluded. This practice has given me the best results, and I cannot remember when I have had trouble with a root which was filled in this way.

*Dr. R. R. Andrews.*—I do not know which method furnishes the most complete obliteration of space in root-canal filling. My simple method works well: after cavities are prepared, I use chloroform, then liquid gutta-percha, then gutta-percha points, then heat with hot air, and pack solidly.

*Dr. Foster.*—I am of the opinion that the complete obliteration of the space in the canal and tubuli is never fully accomplished. I am under the impression, from clinical experience, that the filling-materials best adapted for this purpose are chloro-percha or chloride of zinc and salol. The method of filling is nearly the same with such materials,—a complete isolation of the canal from the fluids of the mouth, thorough dryness of the canal, and, when salol is used, the tooth to be heated externally by the use of an instrument made this shape [see illustration], and heated over an alcohol lamp; when hot, place on the tooth and hold in position until the patient complains of the heat as almost unbearable; then fill with salol by pumping it into the canal with a broach. Capillary attraction will aid its introduction.

*Dr. J. Taft.*—In reply to this question, it may be said that every canal should be thoroughly cleansed and formed with the least practicable loss of material to best facilitate the introduction of whatever material is used.

There are several methods of filling these canals by which all the space can be filled. It is well, after the preparation of the canal, that it should be closed at the end of the root as nearly as practicable; then form a cone-shaped piece of lead, or even a fine-grained piece of wood saturated in some antiseptic fluid; carbolic acid or creosote would serve the purpose. These should be made as nearly as possible of the same shape as the canal, so that upon being pressed in it would fill it, without, however, great pressure. When this cone-shaped lead or wood is prepared, it may be coated upon the surface with oxyphosphate of zinc, and then pressed firmly or driven lightly into the cavity, cutting off any portion that may protrude from the canal or pulp-chamber. By this method not only would the space be completely occupied, but the open ends of the tubuli would be filled. Of course, other materials than lead or wood may be used, as gold, silver, copper, or tin. None of these are better than lead or wood. By this method the canal will be absolutely filled.

*Dr. Truman.*—I am asked to reply to two questions. First, Which method of root-canal filling furnishes the most complete obliteration of the space?

This hackneyed subject has become so exceedingly wearisome that I might be pardoned for tersely stating that no method will obliterate all canals, and it is believed this would be strictly true. It will in the main be true of the central canals, and is always true of those minute canals permeating the dentine rarely or never taken into consideration. The question, however, does not embrace this more extended view, and hence we are left to the question as stated.

There are a great number of filling-materials that will fill the space, if that be all that is required. In ordinary canals gold answers an excellent purpose, and it cannot be gainsaid that some who practised the method in the first period of root-filling, of whom the writer was one, were quite as successful in results as at the present time. But, then, gold or any metal cannot be forced into the minute canals for any depth, as in the superior buccal canals of molar teeth or the anterior roots of the inferior. We are then left to other materials, and, for the same reason as applied to the metals, must discard cotton. The plastics are alone left, and the recently-advocated agent, salol, melting at a low heat. Of the plastic materials, gutta-percha, if made into a semi-solvent solution, would probably fill all non-microscopic canals. There would always be a doubt about it, as there is no possibility of proving the fact. The value of gutta-percha as a filling-material is here left out of the question as not germain to the subject.

The next in importance, if not transcending the previous materials, is oxychloride of zinc. It is by no means certain that this when made in a thin semifluid can be forced into minute canals, but it would probably do this better than gutta-percha.

If all that has been attributed to salol be true,—that when melted it will run by capillary attraction into the minutest canals and then speedily harden,—we have at our command the best material. The question is, Do we know this to be true? Extended experiments out of the mouth in freshly-extracted teeth placed in exactly the same position as in the mouth will alone settle the question. All the processes known as wood filling, gold-wire filling, etc., may be left out of the question, as they cannot, strictly speaking, fill anything; also agents used on cotton or asbestos fibre, such as the balsams, as they cannot reach minute canals.

It thus seems to me, in considering this question, that, for the mere purpose of filling minute canals, salol promises better than any other material. If this were all that is required in canal filling we might rest content; but it unfortunately is not all. Indeed, in the opinion of the writer, it is the least important of the points to be considered.

It almost reaches the point of absurdity to query as to what is best to fill the central canal when the organic matter in innumerable canals is left to the tender mercies of the germs of putrefaction and eventual discoloration of the teeth. As salol is not expected to do more than fill the main canal, and in a degree render it aseptic, that agent which will do more than this—not only fill the canal possibly to its minutest ramifications, macroscopically considered, and then by its coagulating properties reach the organic contents of the tubulated structure—must be the best; and I therefore hold that there is nothing superior to chloride of zinc, for it will, as far as known, quite effectually fill the canals and coagulate all dead material in the dentinal tubes.

*Dr. Ives.*—For the last ten months I have discarded everything in root filling for the following: First, accurate measurement of the root-canal; second, the use of a copper wire; third, the preparation of pure beeswax in a water-bath, to which is added any antiseptic you use,—iodoform, if you wish. I take a thread of the beeswax, and with a spatula roll it down to an absolute point. This, fastened onto the end of a nerve-instrument I pass up as far as possible. With an Evans root-dryer, heated, I drive the wax into the tubuli, adding wax till the canal is full, then with my heated copper point, which protrudes slightly into the pulp-chamber, I send it right to the end. I know that every part of that root-canal is absolutely filled. What space there is between the side and the copper point is filled with the beeswax. Beeswax does not expand nor shrink, and it is not affected by acids or alkalies. I have had great success with it, and have discarded everything else for it.

ANSWERS TO QUESTION 2.

"What is the surest treatment to secure root-canal sterilization?"

*Dr. Jarvie.*—The two questions that are presented for our consideration this evening are of such importance that if they are answered rightly, and the answers of such a character that the *modus operandi* can be put into practical operation and carried into effect by the average dentist, much time and suffering will be saved by the public, and much trouble, annoyance, and discomfiture by the dentist; for no class of teeth with which we have to deal causes so much pain to the patient and so much dread and uncertainty and perplexity to the operator as that which requires "sterilization of the pulp-canal" and "complete obliteration of the root-canal by filling."

The question, "What is the surest treatment to secure root-canal sterilization?" is the one given to me upon which to speak; but the time for preparation has been so short that I shall not attempt an exhaustive treatise upon the subject, but content myself by placing before you, in as simple and as brief a manner as possible, the process I employ and the agents I deem best fitted to bring about the desired result.

The word "sterilization" which occurs in the question would imply that germs are already in existence, and the pulps in a state of putrescence, in the class of root-canals the treatment of which we are to discuss this evening, thus eliminating from the discussion that class of root-canals from which recently devitalized pulps have just been extirpated.

In treating the class of teeth in question, such a course must be pursued that not only the root-canals shall be completely sterilized, but that the contents of the tubuli shall be sterilized also, otherwise the contents of the tubuli may become decomposed and form gases which, having no outlet into the pulp-canal, may force their way out through the cementum, and thus become a constant source of irritation to the pericementum and a subsequent cause of the loss of the teeth. It is seriously questioned by able pathologists whether there is organic matter enough in the tubuli to bring about any such result, but as long as there is the slightest doubt in the matter we should so treat canals and tubuli as to reduce danger from this source to a minimum.

The conditions existing in the root-canals of teeth requiring antiseptic treatment are so different from the conditions existing in almost every other part of the body that treatment which would be proper and successful everywhere else fails utterly in root-canals. In wounds, burns, abscesses, tumors, and in almost all septic conditions the parts are more or less readily accessible; large quantities of the antiseptic may be used as compared to the septic surface, and thus an agent that would be altogether too weak to penetrate and asepticize putrescent matter found in root-canals would be successful. An antiseptic with penetrating qualities powerful enough to permeate putrescent pulp-matter to the uttermost end of a small root-canal has such injurious effect upon tooth-structure as to render it useless for our purpose; therefore thoroughness of mechanical or surgical removal of all putrescent matter in the canals must be depended upon for success rather than for therapeutic effects.

To remove all putrescent matter, use paper points, barbed broaches, shreds of cotton, etc., and as much as is possible enlarge the small canals with square or five-sided Swiss watch broaches. Neither the number of sittings nor the lapse of time is to decide as to the termination of the treatment. Entire removal of septic matter, thoroughness, completeness of this part of the work is the absolute *sine quâ non*, and this can be accomplished at one sitting as well as at a dozen.

With heat, dry the root of the tooth as much as possible. The method I have employed recently, the idea of which I got from Dr. Van Woert, is to roll a piece of copper wire so that the small end is fine enough to penetrate and flexible enough to follow most canals. Insert the wire into the canal with the other end protruding from three-quarters of an inch in the case of a front tooth to possibly two inches in the case of a back tooth. Heat the bulb of an Evans root-dryer, having first removed the point, until it is quite hot, and then insert the protruding end of the wire into the hole in the bulb. Copper being a good conductor, the heat from the bulb will be conducted to the extreme end of the wire, and you will thus get a condition of dehydration in the root-canal and tubuli such as cannot be obtained in any other manner that I am aware of. This method of heating the root-canal is much superior to the ordinary one of a blast of hot air from a syringe, a method by which the crown of the tooth is in danger of being broken by excessive dehydration, while the hot air penetrates but a very short distance into the root-canal, where the dehydration is desired.

The root-canals being now freed from septic matter and the tubuli and canals as dry as possible, they are in the best condition to receive and absorb the antiseptic selected to be applied.

Accepted authorities differ as to the best agent for this purpose, but I think nearly all will agree upon one of the following four as the very best,—viz., carbolic acid, chloride of zinc, oil of cinnamon, and oil of myrtol. The experiments of Professor Miller, of Berlin, give the penetrating properties of the first three in the order I have placed them. I do not know that he has experimented with the oil of myrtol, but he places carbolic acid and the chloride of zinc ahead (in this property) of any of the essential oils. Professor Harlan places a high value upon the oil of myrtol, and prefers it to any other antiseptic as a root-canal dressing, claiming that the essential oils are much superior to any coagulating agent; in fact, denying the use of any coagulant, claiming that the coagulum formed at the end of the tubuli next the canal prevents the entrance of the antiseptic, and so permitting the contents of the tubuli to continue septic, with all the consequent evils.

On the other hand, Professor Kirk has apparently demonstrated that the coagulum found at the mouth of the tubuli becomes a medium by osmosis, for the penetration of the tubuli by the antiseptic.

In this condition of things, where two men, so eminent, each apparently proves the other wrong, each one of us must rely somewhat upon the clinical result of the observation in his own practice. I have been successful with carbolic acid; but if the preparation of the canal has been thorough up to this point, I have no doubt but that you will be quite as successful with the oxychloride of zinc, or the oil of cinnamon, or the oil of myrtol.

Now, gentlemen, I know that it is much easier to write how to remove all septic matter from root-canals than it is to do it. I know, too, that in some cases it is an utter impossibility; but I know, also, that the nearer we approach the perfection of the treatment I have attempted to describe, the nearer we will have complete success in results.

Dr. Darby answered the question by stating that he used sulphuric acid and hot air (bicarbonate of soda after the acid).

*Dr. Louis Jack.*—My answer to this must require the subdivision of cases into those freshly devitalized and those which from any cause have become infected.

In the first class my reliance has been to freely use aristol to saturation with oleum gaultheriæ, and to fill the canals as soon as possible. I should state this is done as soon as all of the pulp-tissue can be removed.

In the second class the question is not so easy, and no one substance can, in my understanding of the subject, be always depended upon. To well consider cases of this kind, they should be arranged in several classes, to meet the various conditions which are presented. The most important consideration is not to force the infectious matter contained in the canal through the foramen, and to avoid the use of infected instruments. I consider it safer to oxidize the contents of the canals with permanganate of potash, by inserting a small crystal, and permit it to decompose the organic matter present, and afterwards to carefully cleanse the canals, following with aristol and oleum gaultheriæ in cases where pus is not present.

Should pyæmic conditions exist, my reliance is upon zinc chloride five to ten grains to one drachm of water, or formalin five-per-cent. solution, in all cases following with aristol as before.

I am careful to avoid the use of irritating medication, and believe better results follow the above line of treatment than when more active remedies are employed, such as pure carbolic acid and corrosive sublimate.

The larger my experience grows the more I am convinced that the greater difficulties of sterilization are neutralized by the lack of the perfect cleanness required at all stages of the treatment. The force of this principle was impressed early upon me by the rules of Dr. Maynard in the treatment of devitalized teeth. His major consideration was that surgical cleanness was of the highest importance, and after this the perfect obliteration of the root-canals. In the treatment of this class of cases Dr. Maynard was supereminent. His results were produced by the greatest care in the treatment, and the utmost patience in the subsequent closures.

*Dr. E. C. Kirk.*—By the application of (1) a saturated solution of sodium peroxide, and (2) twenty-five per cent. pyrozone.

*Dr. A. W. Harlan.*—Repeated washings of the root-canal, first, with neutral peroxide of hydrogen or pyrozone; second, with cinnamon-water or peppermint-water. Dry the interior and introduce a strand of silk or cotton wet with myrtol; seal this in the cavity for one or two days with gutta-percha. After this is taken out, do not let saliva or water get into the root. Fill it.

*Dr. C. N. Peirce.*—For sterilization there are a number of antiseptics which work with almost equal efficiency. Electrozone and pyrozone have both done good service; but for thoroughness in removing all decomposing organic matter there is nothing which goes to work with a determination to clean out everything which is impure as does the sodium peroxide. I have no hesitation in putting a minimum portion of this in the root, and, covering it over with gutta-percha, let it revel for twenty-four hours; then I wash it out with dilute sulphuric acid.

*Dr. S. H. Guilford.*—Root-canal sterilization can, I think, be best secured by first removing with a drill (where possible) a portion of the most infected dentine bordering the canal, and then subjecting the remaining portion of the dentine to the action of some sterilizing medicament that will be rapid in its action, efficient, and non-injurious to tooth-structure.

Mercuric bichloride, while it stands first in the list of germicides in laboratory experiments, has fallen far short in its efficiency in actual practice. Carbolic acid has produced better results at the hands of the majority of practitioners than many other agents with a higher laboratory record; while iodoform, which Professor Miller claims is not a germicide at all, has at the hands of the writer produced better results as a root sterilizer than any drug he has ever used.

*Dr. John S. Marshall.*—First, thoroughly remove all pulp *débris* by broaches wrapped with fibres of cotton. Second, disinfect with ninety-five-per-cent. carbolic acid and dry the canal. Third, flood the canal with absolute alcohol and evaporate with hot air until thorough desiccation is obtained. I succeed best by these means. The rubber dam is indispensable in all cases.

*Dr. McQuillan.*—In treatment of infected canals I have had the best results from the use of sodium and potassium, followed with twenty-five per cent. pyrozone, three per cent., and thoroughly drying with hot air. I have never had so much satisfaction as in the last two years, when I first began the use of this method, which was suggested by Dr. Shreier, of Vienna.

*Dr. Littig.*—Thoroughly cleansing and drying. The kind of drug employed makes but little difference as to whether it is a coagulant or not.

*Dr. Crawford.*—The surest treatment to effect root sterilization is first to isolate the tooth by the use of rubber dam; dry the cavity and as much of the canal as possible before proceeding to cleanse the same of foreign contents; in the first explorations be certain to avoid too much pumping with medicated swabs. *Use bichloride of mercury not stronger than one to three thousand* to frequently wipe out the canal during the operative procedure, so as to keep sterilizing all the newly-infected particles that may occur. When the canal is thoroughly cleansed and made perfectly dry, close it up for a few minutes with a dry tampon of cotton to give the last wiping of bichloride of mercury time to act upon any bacilli or micro-organisms that may be present. If it is desirable to test the condition of asepsis thus produced, I would recommend the thorough packing of the canal with cotton saturated well with pure *German beechwood creosote*, as this is the best antiferment that has ever been placed in the canal of a pulpless tooth. In addition, it certainly has some anodyne effect upon the peridental membrane when it is coincidentally involved as a result of a septic condition of the contents of the pulp-canal.

*Dr. Black.*—Pack with oil of cassia or some mixture containing it in sufficient proportion, not less than one-sixth. The 1, 2, 3 mixture is my standard. Close the cavity perfectly with gutta-percha,—no cotton or gum,—moistening the walls with eucalyptus first, and let it remain one week. Put on the dam before beginning and before removal at the end of time.

*Dr. Crouse.*—I generally use this method: when the pulp has been dead some time and the tooth needs sterilizing, I use what I think is called Black's 1, 2, 3 compound, composed of one part carbolic acid, two parts oil of cinnamon, and three parts oil of wintergreen. Oil of cinnamon will sterilize a tooth quickly, but should not be used for the front teeth, as it frequently turns them yellow. For teeth in the front of the mouth I use bichloride of mercury, or carbolic acid, or both; the bichloride first, and then the acid. After applying arsenic I generally remove the pulp in two or three days. This can be readily done by wrapping a little cotton around a small, smooth broach, forcing it into the canal, and twisting the broach slowly, when the pulp will usually cling to the cotton, and thus be removed entire. After this I fill immediately, using the above-named medicine before putting in the oxychloride of zinc filling.

*Dr. Truman.*—I suspect the best reply to make to this would be, I don't know. The means for effecting sterilization in anything are as yet in their infancy, and any dogmatic statement that this or that method will effect it must be taken with a large allowance for error.

The question is not free from ambiguity. It is difficult to know whether sterilization is meant to include the time just previous to filling, or to prevent the remote and very possible element of danger that may arise subsequent to the filling. It is assumed that it is intended to apply to the first-named condition, and upon this understanding it will be considered very briefly.

As the main canal is alone in question, it is very plain that we are left to the agents that effectually inhibit pathogenic germs. I do not use the word destroy here, for I have no positive proof that germs can be absolutely destroyed upon a vital tissue and it retain its vitality. The antiseptics in use in dentistry are limited, but in my opinion no reliance should be placed upon a single one of these, but the treatment should be by a continuation of agents and methods. The importance of sterilization to the fullest extent possible cannot be overstated.

To intelligently consider this question, the condition of the root should be taken into consideration. All canals are not equally charged with decomposed matter; but, as this is an uncertain quantity, they should be given the full benefit of the existing doubt and treated exactly alike. This involves, first, thorough cleansing of the canal; second, equal thoroughness in making it aseptic.

The agents used may be briefly stated to be,—

1. Those that act on organic matter,—hydrogen dioxide or pyrozone; or the alkalies, as sodium peroxide; or a combination of the escharotics and alkali, as the process recommended by Dr. Callahan of sulphuric acid and bicarbonate of soda. These are practically cleansers, while in a degree they are antiseptic.

2. Follow with one of the following antiseptics proper: mercuric chloride, hydronaphthol, thymol, creolin. The latter I regard as one of the best antiseptic cleansers we have in use. Used in full strength with cotton to wipe out the canal, it is most effective. If this has been carefully done, the canal should be dried by the warm-air blast. This, in my judgment, is the proper treatment of the canal, and will place it in the best possible condition for subsequent filling, if the minute microscopical cells are not to be considered. If these are regarded of vital importance, as unquestionably they are, then the filling as recommended in answer to question first, oxychloride of zinc, must, in my opinion, be adopted, as I place no reliance in the lasting effect of any of the essential oils.

*Dr. Taft.*—The treatment of any pulpless canal should and ought to be regulated by the conditions that may be present. A canal from which a healthy or comparatively healthy pulp has been removed, when cleansed, is in the best condition for filling. No sterilizing nor antiseptic treatment is indicated if the tooth has been protected against moisture and the entrance of foreign substance. Medication of such canal is likely to be attended with more injury than benefit.

The question of coagulating treatment to act upon the contents of the tubuli has excited some discussion. The introduction of coagulation into a thoroughly cleansed and prepared canal will not, perhaps, in any case be the occasion of injury. The extent, however, to which it may be operative, will depend upon the character of the agent and upon the condition of the structure to which it is applied. Greater penetration will be effected in some teeth than in others. It is a question not yet decided as to whether such coagulation accomplishes any benefit or not.

Canals in which pulps have been destroyed by disease are presented in very different conditions. In some the remnants of desiccated pulp remain, while in others the entire structure of the pulp is broken down and the canal filled with pus or sanies. With a desiccated exudate through the canal, simply cleanse by manipulation and wash with warm water or some simple dilution; then, after drying the canal, some antiseptic agent may be employed.

Sulphuric acid treatment, brought prominently to the notice of the profession within the last two years by Dr. J. R. Callahan, is in very many cases of pulpless canals an excellent treatment. By its introduction it facilitates the removal of all *débris.* It also acts upon the walls of the canal, dissolving from the surface more or less according to the strength and amount of the acid used, thus enlarging the canal instead of with an instrument. After the employment of this mode of treatment no further sterilizing agents need be used. Every organism reached by the acid would be effectually destroyed.

In all favorable conditions canals should be filled immediately after their thorough cleansing and proper forming. In the less favorable cases expectant or test treatment in the way of thorough closure with some easily removable substance, such as gutta-percha or Hill's stopping, may be employed, this remaining for a few days to a few weeks according to the more or less favorable conditions that are apparent.

*Dr. Andrews.*—I do not know the surest method of canal sterilization. My method, though very simple, works well: clean thoroughly, use five-per-cent. pyrozone, dry thoroughly, and then fill, having all instruments surgically clean.

*Dr. Foster.*—Bichloride of mercury, or cyanide of mercury, or chloride of zinc.

*Dr. Hart.*—I do not think that Schreier's preparation of sodium and potassium will ever become popular in general practice. We may use it now and then, but it is a preparation that takes longer to apply in any canals than the medicaments that we usually employ. I see that one gentleman recommends leaving it in the canal and sealing it up. I can scarcely understand what advantage would ensue, because it acts so quickly that immediately upon its being placed in any canal where there is moisture the effect is obtained; the bubbling of Schreier's preparation is immediate, and I think there is a distinct disadvantage in leaving it there after the effervescence has taken place. One might summarize what we have heard to-night and come to the conclusion that cleanliness is the principal thing. We have heard praised nearly every therapeutic agent that we use to obtain asepsis in a root-canal, and no doubt each one is successful after the proper cleanliness has been obtained. In my own practice, after using bichloride of mercury, 1 to 3000, for some time, I went back to the old standard, carbolic acid, and have used that ever since. It may have been from the method I used, from pumping too hard in the canal, but I do not think that was it, because I used the same care that I do with the carbolic. I do not dread the coagulating influence of the carbolic acid. Whether it is that the subsequent asepsis is induced by the osmotic action or not I cannot say, but I have had least trouble with the carbolic acid.

*Dr. Merriam.*—Dr. Whitton, of Boston, whose early death is, I believe, the greatest loss that Boston has had in my day, gave this subject a great deal of attention. One part of his practice that I have not heard mentioned in connection with the subject to-night was always to remove by a rotary motion. An evil that exists in the schools is the probing by students; the desire of the student to see how far he can get when he first opens the tooth, perhaps to satisfy himself or to excite the admiration of the patient, is natural, but the passing of an instrument through a putrid mass is certainly a most dangerous thing. Dr. Whitton's method was to wind cotton carefully on small broaches, having as many as needed ready for each case. He removed carefully from the pulp-chamber, and as fast as the cotton on one broach became soiled he took a fresh one, and spent all the time necessary at the first operation in cleaning the root. He filled a great many of his cases immediately, and, as nearly always follows, I think with any one who adopts a system and gains the confidence of the patient, he had very excellent results. One thing about the peroxide which we mentioned to-night, I think we overestimated it and its effect on the tissues. We should think of it as a detergent, and to the extent that it is a detergent it is of benefit; but it has no effect on the tissues themselves. At Billings & Clapp's, Boston, who make the best peroxide I have used, they were kind enough to tell me that it was best to buy it from the nearest manufacturer. I have followed that hint since, going right to the factory and getting what I need. We are all very much better off than formerly in the instruments that we have. The music wire that has come to our assistance allows us to make instruments and to use them with better security than anything we had before.

*Dr. Hodson.*—I am fully persuaded that perfect cleanliness is our sheet-anchor in the premises, and the most perfect elimination of all septic material the very first necessity, the methods are individual. My own plan is never to touch putrescent contents of the canals upon first opening to them, but apply full disinfection under a cotton and wax dressing for twenty-four hours, and even then am specially careful, after very gentle use of the bare broach for the mass, not to push anything (even air, which in the circumstances would be poisoned air) through the ends of the canals, which I am persuaded any swabbing out process will surely do, but I alternately fill the canals with warm water by syringing, and then draw it loaded with putrid material down and out by capillary attraction through the insertion of the paper points, which I presented to you twenty years ago (and which, by the way, another man has applied a little extra stiffening material to and boldly advertises the whole as his own). This is a *pulling process* entirely, and I cannot but feel it to be, to me at least, of real value in the premises. This principle of capillary attraction I employ in many directions in my general work, and find it of real value.

*Dr. Sailer.*—One point of our discussion which has not been developed very largely, and at which I have been somewhat surprised, is the nerve instrument. Dr. Guilford alluded to the drill, but I would rather call it the nerve-canal drill. I think Dr. Guilford has struck about as clear a way of sterilizing a root as we can possibly get at. If you cut away the dead matter you certainly sterilize it. You can then use the cleansing medicament, and cleanse out what is left. I always follow the process of using a canal-drill and taking out the dead matter and enlarging the canal as far as I can, then, as we heard to-night, avoid carrying the dead matter through. You can always avoid that by using a hook instrument made by Donaldson, using a large one first, and reducing it gradually until you can just get to the end of some of the roots,—not all, of course. You will not have much trouble if you do that. I want to call attention particularly to the nerve-drills. To my mind, those are the most important instruments in sterilizing the canal. I have very frequently drawn out a putrid nerve by the use of a small nerve-drill. In doing that, you want to use a drill that is very much smaller than the nerve itself. I do not mean this for interior roots of lower molars or some of the inferior teeth, nor for the buccal roots of superior molars.

*Dr. Brockway.*—The first step in the sterilization of a root-canal is to get as free access to it as possible, cutting away at the *outset* all that you intend to. For the removal of the contents of the canal, I make much use of the Morey or Gates-Gliddon root-drill, while using them having my assistant at the same time pump into the canal from a syringe water as hot as can be borne; this washes out the *débris* and does much towards cleansing the root.

I know that some of my brethren condemn the use of the root-drills as needless and dangerous, but I find them most efficient if used with skill and judgment, always taking the largest sizes *first* to avoid breaking. For the sterilization of the canal I make use of the various disinfectants, bichloride of mercury, peroxide of hydrogen, peroxide of sodium, etc., as seems to be indicated. For the past two years I have made much use of Schreier's preparation of kalium-natrium, and with great satisfaction. Where the canal is filled with exceedingly putrid matter I often apply it before making any attempt at removal. This renders the operation much less disagreeable. My experience with this preparation has been that it is one of the most efficient and expeditious that we have, its action as you know being almost instantaneous.

Having the canal clean and sterilized, I think it makes not much difference what antiseptic is used for further treatment; there are many good ones in common use. I rely mostly upon oil of cinnamon in conjunction with the root-dressing devised, I think, by Dr. Lord.

I omitted to mention the use I make of the Evans root-drier in the sterilization of root-canals. It is exceedingly valuable in this respect. I think no one has spoken of it except Dr. Jarvie, but it should be in general use. Through its flexible silver point one can safely heat the canal so as to destroy any germs that may be present.

I have made use of the method spoken of by Dr. Jarvie in some thirty or forty cases, and so far am much pleased with the results.

*Dr. Hill.*—It is rather pleasant to hear our most eminent men agree very nearly in the treament that most of us use, and it shows that we have brought down the treatment of pulpless teeth in various forms pretty nearly to one that we all use. I would like to tell my experience with drills, but I have not used them. Where I did not want them they were very useful, but where I really wanted a drill, and ought to have had one, I could not have made use of it, for I never could find a straight one that would bend and go around a corner. I do not want to be misunderstood. I have not in twenty years been without sulphuric acid, and I do not think I have ever treated an abscessed tooth without sulphuric acid, 20 to 50. Dr. Callahan's idea is a different method. I have never tried it. After treating diseased roots of teeth where there have been abscesses, there is always trouble in such cases, and you want to get to the end of the root. I clean out a root with five or six fibres of cotton on a broach, allowing the cotton to extend one-half to three-quarters of an inch onto the broach; then I turn it right around the broach until I have a perfect screw made of cotton. Make it small or large, but work onto the end of the root until you have it very small, and you can get every particle of foreign matter out of the root. Take an abscessed tooth, and if the root is decayed put a forty-per-cent. solution of sulphuric acid there, and the odor is something awful. You can clean it out with soda or warm water as you choose. Where there is an abscess on the root I always pump that through. If the processes are diseased at all, it will hasten the death or dissolve what is there. I have used sulphuric acid in that form ever since Atkinson first introduced it. I have tried to find the date when it was first introduced, and I think it was by Lister in the London *Lancet* some time in the seventies. When Callahan's treatment came out I was surprised that more dentists did not use that method. There is nothing that will clean out a root so nicely or thoroughly as that. I am one of those who never fill a diseased root at the first or second sitting. At one time I used peroxide of sodium to have it work out through the dental tubuli.

*Dr. Merriam.*—I have been lately experimenting in rather a large way to see what we could do in the improvement of instruments by beginning at head-quarters. I wrote to one of the large steel firms in regard to music wire, and had them make for me that wire in octagon forms. It is of such temper that it can be readily filed and flattened without changing the temper in any way. I brought some of this steel with me to New York and left it at Schmidt's. They made a few instruments for me this afternoon, and if you will accept them as a report of progress I should like to show them to you. I would speak also of the milliner's needle. It is very long and narrow and the tapering is very slight. The temper can be drawn as stated by Dr. Perry, and, fitted into a round handle, it makes a nice instrument for exploring. They can be obtained in very small sizes indeed. The steel wire I spoke of can be made into the various forms that a dentist wishes. One of the ideas I had was that the small octagon would do away with the present strain; the force used is often too much for the point.

*Dr. Turner.*—I think it would be well to call attention to a method of filling root-canals recently introduced by Dr. Van Woert. It consists of filling them with paraffine and iodoform. Dr. Jarvie spoke of Dr. Van Woert's method of treating the canals with the Evans root-dryer. After the canal is dry some of the paraffine is introduced into the cavity around the copper wire that extends into the nerve-canal, and the heat from the Evans root-dryer melts this immediately, and by capillary attraction this runs into the root-canal and fills it absolutely. The wire is withdrawn while hot, and the paraffine remains in the canal.

*Dr. Hill.*—Of course, everything that Dr. Perry reads is almost perfect. I am delighted with the paper, and I am not at all surprised at its being so perfect coming from him; but I wonder why Dr. Perry made such a preparation of handles on the broaches. Swiss broaches come from the size of a hair, and run from about two and a quarter inches up to five inches long. I have never used anything else. I never had a Donaldson broach or any of the others in my office. Up to a very few years ago I barbed all my own broaches. Take an instrument two and a half inches long, say nearly an inch of it being handle, and, for all purposes of putting on cotton and rotating it, it is far preferable to having an extra handle on it. If I understand the broaches as I see them there, you can just as well have the long ones. I find fault with Dr. Perry's way of drawing the temper. He puts the instrument in a glass-tube and draws the temper equally. I think that is a mistake. You light your little lamp, hold that just above the guard which is on it, commencing just at the end of the broach, anneal it just enough to get the blue, and you can draw the temper of the broach perfectly, having the broach stiff near its handle, and at the sharp end you can have it perfectly pliable. I have nothing whatever to say in regard to filling roots. We all do about the same thing. Nearly all of us use chloride of zinc in some way. We may have different ways of applying it. In filling the roots Dr. Perry has the gold wire, which is undoubtedly a good thing. Just as good a thing, or better, is to wind the foil on a fine broach, then pull your foil a quarter of an inch beyond the end of the broach and roll it in your fingers, and you will get as fine a point as possible. For all incisor teeth it is a very good way of filling. There is nothing better for a single-rooted tooth than gold; in that way it can be done very readily. I must congratulate the Society upon getting these various opinions together in the form they have. It is very valuable. I do not think it has ever been done before in any society. This ought to be somewhat conclusive in regard to the matter of sterilizing the roots of teeth and filling them.

*Dr. Perry.*—Dr. Maynard had a strong preference for wood handles on all his delicate instruments. He claimed he could have more delicacy of touch with a wood handle than he could without it. It may be that unconsciously I have imbibed a little of his feeling, and for many years I have had my instruments made with the handles of a light kind of wood. I was aware that there were broaches made with longer handles than these. I use many of them without the handles, of course. I do not use them with the wood handles in all cases. There are many operations far back in the mouth where you often need a little handle on the instrument. There is nothing original claimed in reference to those broaches or their use, and as for filling the canals with gold on the end of the broach, you have all done that I suppose. I abandoned that many years ago for several reasons, because it takes a great deal of time, and after it is all through you have a filling that you cannot get out, and it is not an antiseptic material. It may happen that you may never want to take it out, but perhaps you might want to do so, and then there would be trouble. I will defy anybody to fill these little roots with more success than can be done with these wires. That is a great deal for me to say, but I feel sure that I get that little point of gold right down to the very end. I carry with it just enough sterilizing matter to fill up the space and check the trouble at its very seat. You must have some substance to carry before the instrument to get it tight. I know it is perfectly practicable with these little points of gold, for they are prepared in a moment. You can have them on hand, pick them up, moisten them with chloro-percha, and it is done in an instant. I do not care what you use, whether it is chloro-percha or oxychloride of zinc, or this preparation of Dr. Van Woert, which Dr. Lord has used so long, but you are very liable to forget that in those closed ends you get the air in, and think you have filled them and you have not.

*Dr. Hodson.*—I was just about to speak of that particle of air. I am accustomed to using chloro-percha with the gutta-percha points following, and employ the same capillary principle of which I previously spoke, by first filling the canal with clear chloroform, or with so little gutta-percha in it as to be *as limpid as the clear* chloroform, and then placing a very tiny, smooth broach wet with it in the nerve-canal and reaching to its end, being sure, of course, that an opening is left along the side of the broach so that capillary attraction can act. I drop the chloro-percha at (not over) the entrance of the canal, and the capillary attraction, acting both over the wet broach and the wet canal, carries it surely to the end of the root. I am certain of that by the pumping process proving it. Then I insert the gutta-percha point and carry it gently but quickly to its seat in the canal, as before stated.

*Dr. Jarvie.*—In the answers from different gentlemen the use of certain waxes is advocated. I think the process described by Dr. Turner is exceedingly valuable. Perhaps further experience may change my mind, but at the present time I think it is the best way we have of filling root-canals. The method is to prepare a piece of copper wire small enough to penetrate the finest root-canals and graduated in size to fit them. Fill the cavity of decay, or put into it a sufficient amount of paraffine mixed with iodoform. Heat the bulb of an Evans root-dryer and let the protruding end of the wire enter the hole in the bulb. The heat is conducted to the extreme end of the wire, and the paraffine is melted. Draw out the wire and capillary attraction will draw the paraffine into the space left vacant by the wire. You can see the entire process by experimenting with glass tubes. It is marvellous how the wax will fill up the space previously occupied by the copper wire. You can make the copper wire as fine as the steel wire, but of course when it is very fine it loses heat quickly. You must have the wire according to the size of the root-canals. I think the system of Dr. Perry for the fine canals is very good, indeed, but for the larger ones I think this method of Dr. Van Woert is better.

Adjourned.

John I. Hart, D.D.S.
*Editor New York Odontological Society.*

## Figures and Tables

**Figure f1:**