# Erratum

**Published:** 1895-10

**Authors:** 


### ERRATUM.

On page 565 of the September number, eighth line from bottom, "one drachm of water" should read "one ounce."

